# Interstitial lung disease associated with drug therapy

**DOI:** 10.1038/sj.bjc.6602063

**Published:** 2004-08-31

**Authors:** P Camus, S Kudoh, M Ebina

**Affiliations:** 1 Services de Pneumologie, University Hospital and Medical School, Université de Bourgogne, Dijon, France; 2 Fourth Department of Internal Medicine, Nippon Medical School, Tokyo, Japan; 3 Respiratory Oncology and Molecular Medicine, Institute of Development, Aging, and Cancer, Tohoku University, Sendai, Japan

**Keywords:** gefitinib (‘Iressa’), NSCLC, chemotherapy, interstitial lung disease

## Abstract

Drug-associated interstitial lung disease (ILD) is not uncommon, with diverse patterns ranging from benign infiltrates to the potentially fatal acute respiratory distress syndrome. As acute respiratory failure due to drug-associated ILD has an unpredictable onset and rapid time course, establishing a diagnosis is often difficult. An accurate diagnosis is based on clinical, radiological (including high-resolution computed tomography) and histological manifestations, although is often only possible by exclusion. Cancer chemotherapy is commonly associated with acute disease that, on pathology, is often diffuse alveolar damage. Furthermore, a combination of drugs with or without radiotherapy can increase the risk of ILD. This article reviews treatments for non-small-cell lung cancer (NSCLC) that are associated with the development of ILD and how systematic evaluation of the possible role of these drugs in ILD is warranted. A difference between Japan and the rest of the world in reporting rates of ILD when gefitinib (‘Iressa’) has been used in advanced NSCLC is also discussed. However, the difference remains unexplained, leaving important epidemiological and mechanistic questions.

## HISTORY AND CLASSIFICATION OF DRUG- ASSOCIATED INTERSTITIAL LUNG DISEASE (ILD)

Drug-associated lung disease is common, with infiltrative lung disease comprising 70% of cases. Recent experiences of the approval and introduction of the novel cancer therapy gefitinib (‘Iressa’) for the treatment of advanced non-small-cell lung cancer (NSCLC) have indicated international differences of occurrence of ILD. The frequency of ILD-type events in Japanese patients (1.9%) appears to be higher than in the rest of the world (0.3%). This observation has highlighted the need for greater understanding of the characteristics of drug-associated ILD. Here, we describe the usual forms of drug-associated ILD and methods of diagnosis and pathology. The experience of ILD in Japanese patients treated with gefitinib is illustrated in detail and includes four detailed case reports to aid recognition.

There are many patterns of drug-associated ILD, ranging from benign infiltrates to life-threatening acute respiratory distress syndrome (Camus *et al*, 2001) (Table 1

**Table 1:** Acute and ‘classic’ drug-associated ILD

**Drug-associated acute ILD with acute respiratory failure**	**‘Classic’ drug-associated ILD**
*Methotrexate lung*	*Hypersensitivity pneumonitis*
• Typical drugs: methotrexate, chrysotherapy (gold)	• Typical drugs: methotrexate, chrysotherapy, cyclophosphamide, nitrofurantoin, antidepressants
• Characteristic features: lymphocytic BAL fluid, history of infiltrative lung disease, high fever	• Characteristic features: sensitisation, lymphocytic BAL fluid
• Diagnosis: BAL, biopsy	• Diagnosis: histopathology, BAL
• Outcome: favourable, usually recovers with steroids or on dechallenge	• Outcome: favourable, usually recovers with steroids or on dechallenge
	
*Acute EP*	
• Typical drugs: minocycline	• Typical drugs: methotrexate, sulphasalazine, minocycline, para-aminosalicyclic acid, nitrofurantoin, nonsteroidal anti-inflammatory drugs
• Characteristic features: eosinophilic BAL fluid, fever, skin rash	• Characteristic features: eosinophilic BAL fluid, skin rash, fever, ‘photographic negative of pulmonary oedema’ on radiography
• Diagnosis: BAL	• Diagnosis: BAL
• Outcome: favourable on cessation of minocycline	• Outcome: favourable, usually recovers with steroids or on dechallenge
	
*Chemotherapy lung with DAD*	*Amiodarone pneumonitis*
• Typical drugs: bleomycin, busulfan, carmustine, mitomycin	• Typical drugs: amiodarone
• Characteristic features: DAD	• Characteristic features: dyspnoea, chest pain, cough, mild fever, asymmetrical nonsegmental opacities on radiography
• Diagnosis: radiography, HRCT	• Diagnosis: radiography
• Outcome: death in 50–60% of patients	• Outcome: favourable
	
*Pulmonary oedema*	*Pulmonary fibrosis*
• Typical drugs: ARA-C, B2R-agonists, blood, blood products, narcotics, diuretics	• Typical drugs: amiodarone, chemotherapy
• Characteristic features: permeability leakage in alveoli, bilateral consolidation on radiography	• Characteristic features: fibrotic nonspecific interstitial pneumonia
• Diagnosis: water in BAL fluid	• Diagnosis: radiography
• Outcome: favourable	• Outcome: poor
	
*Alveolar haemorrhage*	*Desquamative interstitial pneumonia*
• Typical drugs: oral anticoagulants, fibrinolytic agents, platelet glycoprotein inhibitors	• Typical drugs: methotrexate, interferons, etanercept-D2E7
• Characteristic features: bland haemorrhage or capillaritis, diffuse GGA on radiography	• Characteristic features: alveolar accumulation of macrophages, mosaic pattern radiograph
• Diagnosis: BAL	• Diagnosis: radiography
• Outcome: favourable	• Outcome: favourable

ILD=interstitial lung disease; BAL=bronchioalveolar lavage; EP=eosinophilic pneumonia; DAD=diffuse alveolar damage; HRCT=high-resolution computed tomography; GGA=ground-glass attenuation.

). These clinical patterns differ depending on the patient's illness and drug factors, including the type of drug. Hence, an increasing number of drugs are being recognised as being capable of inducing ILD (Foucher *et al*, 2003). Classification of drug-associated ILD is still emerging and is based on clinical, radiological and histological manifestations. The categories include acute-onset ILD with dense pulmonary infiltrates leading to acute respiratory failure, ‘classic’ ILD, lobar consolidation, migratory pulmonary opacities and lung nodules.

Interstitial lung disease is difficult to diagnose. Clinical, radiological and pathological observations are necessary to exclude other conditions with similar symptoms, such as lymphangitis carcinomatosis, pneumonia, allergy, cardiogenic oedema and pulmonary haemorrhage (Merad *et al*, 1997). Identification of drug-associated ILD is further hampered, as clinical imaging and pathological patterns are not diagnostically reliable; indeed, one study of high-resolution computed tomography (HRCT) and histopathology showed that pathological pattern and imaging did not correspond in >40% of cases (Cleverley *et al*, 2002). A detailed recent drug history is essential in making the diagnosis, but there are important caveats: the rate of onset of the disease may be acute or delayed, and multiple therapies may obscure the responsible therapy's role, while long-term lung-retention of the drug may impair resolution on discontinuation of the drug. A standard of drug-associated disease is the use of re-exposure or rechallenge to confirm diagnosis. However, there is a natural reluctance to put the patient at risk of further illness, particularly if the drug-associated disease is severe. Furthermore, the patient may have comorbid disease that modifies the progression or resolution of drug-associated illness.

The most common histopathological patterns of drug-associated lung injury include pulmonary oedema, diffuse alveolar damage (DAD), nonspecific interstitial pneumonia, bronchiolitis obliterans organising pneumonia (BOOP), eosinophilic pneumonia (EP) and pulmonary haemorrhage. Drugs can be associated with several concurrent pathological patterns. While HRCT may allow prediction of the pathological pattern for ILD in general, this is not the case for drug-associated ILD. Several recent review articles (Ellis *et al*, 2000; Aviram *et al*, 2001; Cleverley *et al*, 2002; Erasmus *et al*, 2002) comprehensively illustrate this point.

### Illustrations of drug-associated ILD with acute respiratory failure

Several patterns of ILD with acute respiratory failure have been observed (Table 1). Acute respiratory failure due to drug-associated ILD generally has an unpredictable onset and rapid time course.

**Figure 1:**
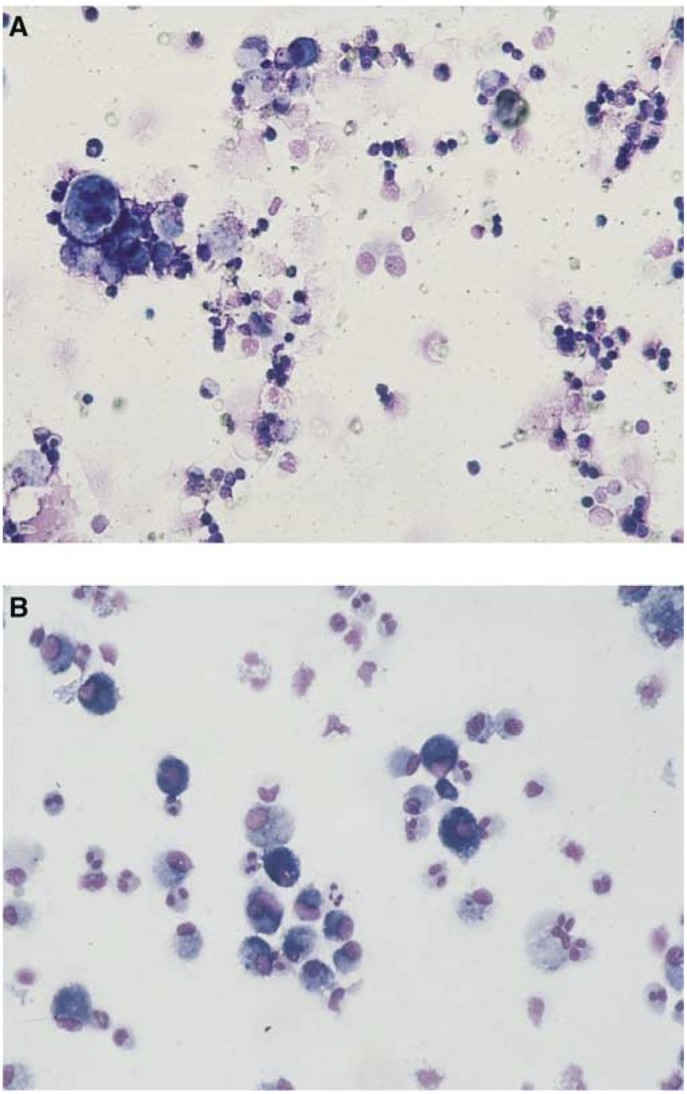
BAL of patients with drug-associated acute ILD. (**A**) Lymphocytes present in BAL fluid as observed, for example, in cases of ‘methotrexate lung’. (**B**) Eosinophils present in BAL fluid as observed in EP due to, for example, minocycline treatment.

‘Methotrexate lung’ can occur when methotrexate is used to treat rheumatoid arthritis or cancer. The pattern is similar to the acute forms of lung disease seen with other drugs. Lymphocytes are observed in the bronchioalveolar lavage (BAL) fluid and lung biopsy reveals inflammatory cell interstitial infiltrates and often demonstrates features of hypersensitivity (Figure 1A

) (Imokawa *et al*, 2000). Chest radiography usually reveals diffuse interstitial or mixed interstitial and alveolar infiltrates (Imokawa *et al*, 2000).

Antibiotic agents such as minocycline, a semisynthetic tetracycline used in the treatment of acne, can cause acute ILD leading to acute respiratory failure due to acute EP (Oddo *et al*, 2003). Eosinophilic pneumonia is essentially diagnosed by increased number of eosinophils in the BAL (Figure 1B) and is often associated with a blood sample eosinophilia; lung biopsy should be avoided. High-resolution computed tomography scans show patchy consolidation with centrilobular opacities.

Most chemotherapeutic agents can cause ‘chemotherapy lung’, acute ILD with the pathological features of DAD. Incidence is estimated at 10%, although it is difficult to obtain accurate estimates due to the complexities of diagnosis and the small patient numbers involved. The risk increases with dose and there may be potentiation by high oxygen and concurrent use of granulocyte colony-stimulating factor. Diffuse alveolar damage is characterised histologically by the presence of alveolar airspace and interstitial oedema, hyaline membrane formation and proliferation of type II pneumocytes. Radiographs show bilateral patchy or homogenous airspace consolidation involving mainly the middle and lower lung zones. High-resolution computed tomography scans demonstrate extensive bilateral ground-glass opacities and areas of airspace consolidation (Akira *et al*, 2002).

In some instances, ILD leading to acute respiratory failure can occur due to pulmonary oedema as a consequence of permeability leakage in the alveoli. Alveolar haemorrhage by haemorrhage or capillaritis can also cause acute ILD leading to acute respiratory failure, typically with oral anticoagulants, fibrinolytic agents and platelet glycoprotein inhibitors.

In summary, acute ILD leading to respiratory failure has an unpredictable onset (from days to years) but rapid development. Bronchioalveolar lavage is helpful in diagnosis, while biopsy can generally be avoided, as it does not always provide a specific diagnosis. Steroid therapy is useful and the overall prognosis is favourable, except in the case of DAD.

### ‘Classic’ drug-associated ILD

Hypersensitivity pneumonitis is probably the most commonly described pattern of ‘classic’ drug-associated ILD. ‘Allergic’ or ‘immunological reaction’ of the lung has also been used instead of ‘hypersensitivity pneumonitis’, as hypersensitivity pneumonitis can be considered as only the result of inhaled materials. It is not the most common form of drug-associated disease and can result from therapy with a number of drugs, including methotrexate, cyclophosphamide, nitrofurantoin and antidepressants (Gonzalez-Rothi *et al*, 1995). Hypersensitivity pneumonitis has a granulomatous pathology, characterised as an immunologically induced disorder resulting from sensitisation.

Acute and chronic forms of EP are well recognised as manifestations of drug-associated ILD. Mild drug-associated ILD EP is most commonly observed in association with methotrexate, sulphasalazine, para-aminosalicylic acid, nitrofurantoin and nonsteroidal anti-inflammatory drugs. Eosinophilic pneumonia is characterised histologically by the accumulation of eosinophils in the alveolar airspaces, with infiltration of the adjacent interstitial space by eosinophils and variable numbers of lymphocytes and plasma cells. Chest radiography and HRCT demonstrate bilateral airspace consolidation, which tends to involve mainly the peripheral lung regions and the upper lobes.

Short-term (10 days) and long-term (14 years) therapy with amiodarone can lead to dose-related pneumonitis, with onset being acute, rapid, progressive or delayed. Radiographic images, although variable, are invaluable in diagnosis. In addition, gradual-onset pulmonary fibrosis, although rare, can develop following therapy with amiodarone, chemotherapy or radiotherapy. Furthermore, additional unusual patterns of drug-associated ILD such as granuloma can be observed following methotrexate, interferon or bacillus Calmette–Guerin therapy.

There are several patterns of ILD other than ILD with acute respiratory failure and ‘classic’ ILD. Exogenous lipoid pneumonia is an uncommon condition resulting from aspirating or inhaling lipoid material found in laxatives or various aerosolised industrial materials (Spickard III and Hirschmann, 1994). These substances produce a foreign-body reaction and proliferate fibrosis in the lung. Diagnosis is easily reached by examination of sputum, which demonstrates lipidotic macrophages. Early discontinuation of exposure leads to lung function improvement before any serious complications have chance to develop.

Drug-associated BOOP can occur following treatment with amiodarone, interferon, mesalazine, bleomycin, gold, nilutamide or penicillamine. Bronchiolitis obliterans organising pneumonia is a chronic inflammatory process, predominantly involving the respiratory bronchioles and alveolar ducts. It is characterised by lipid-laden macrophages in the distal airspaces and focal plugs of immature fibroblasts (Masson bodies) within the airways. Prognosis is favourable, although drug withdrawal is essential. Additionally, multiple lung nodules are observed with bleomycin, amiodarone and minocycline therapy. These lung nodules mimic metastases and are generally poorly defined.

## RECENT EXPERIENCE OF DRUG-ASSOCIATED ILD IN JAPAN

A considerable proportion of the available literature on ILD, both general and drug associated, comes from Japan. Explanations include the possibility that ILD may be more prevalent among the Japanese or that a greater awareness of the disease could lead to more frequent diagnosis. For example, there is comprehensive prescribing information for chemotherapeutic agents with a higher degree of concern regarding ILD. This may account for the more frequent reports of chemotherapy-associated ILD in Japan than elsewhere in the world. Alternatively, there may be a specific increased genetic susceptibility to ILD among the Japanese population.

### ILD associated with conventional NSCLC chemotherapy

Current guidelines recommend the use of platinum-based combination chemotherapy for patients with advanced lung cancer combined with radiotherapy, depending on the stage of disease (ESMO, 2003). In the USA and Japan, the most frequently used protocol is carboplatin and paclitaxel, while in Europe it is cisplatin and gemcitabine.

The literature includes a number of reports of ILD thought to be associated with lung cancer chemotherapy, such as gemcitabine (Lilly, 2003), docetaxel (Kunitoh *et al*, 1996; Merad *et al*, 1997; Wang *et al*, 2001), paclitaxel, irinotecan and vinorelbine (Erasmus *et al*, 2002). Treatment of NSCLC with combination chemoradiotherapy has also been associated with the development of ILD (Aviram *et al*, 2001). Increases in drug dose and combinations of different drugs, and sequential use of radiotherapy and chemotherapy, increase the incidence of ILD.

In recent years, interstitial pneumonia has been described in Japan as an important adverse event of new chemotherapy drugs such as paclitaxel, docetaxel and gemcitabine.

### Experience with novel lung cancer treatments

In clinical trials of gefitinib, an epidermal growth factor receptor tyrosine kinase inhibitor, ∼40% of patients with advanced NSCLC who had received at least one prior chemotherapy regimen demonstrated clinical benefit of response/disease stabilisation accompanied by a rapid improvement of symptoms (Fukuoka *et al*, 2003; Kris *et al*, 2003). This represents unprecedented disease control and symptom improvement in this disease setting. Despite an absence of pulmonary complications in preclinical evaluations and no obvious imbalance in pulmonary complications/ILD in the gefitinib and placebo arms of INTACT (‘Iressa’ NSCLC Trial Assessing Combination Therapy) 1 and 2 (Forsythe and Faulkner, 2003a), a significant number of reports of patients developing ILD surfaced shortly after the introduction of gefitinib in Japan. It recently became apparent that the reporting rate of gefitinib-associated ILD is higher in Japan than in the rest of the world, although overall the balance of clinical benefit with gefitinib outweighs the risk of ILD.

In >92 000 patients worldwide, who have received gefitinib, the reported incidence of ILD is ∼1%; outside Japan, in a global compassionate-use programme involving >39 000 patients, the reported incidence is ∼0.3% (Forsythe and Faulkner, 2003b). In Japan, 39 000 patients have received gefitinib therapy and the reported incidence of ILD is around 1.9% (Table 2

**Table 2:** Incidence of ILD in gefitinib-treated patients (Forsythe and Faulkner, 2003b)

**Gefitinib-treated patients**	**Incidence of ILD (death), %**
Globally (*n*=∼92 750)	0.99 (0.36)
Japan marketed use (*n*=∼39 600)	1.86 (0.69)
Outside Japan (*n*=∼53 150)	0.34 (0.11)
US EAP (*n* =∼24 200)	0.39 (0.08)
Rest of world EAP (*n*=∼15 000)	0.38 (0.21)

EAP=expanded access programme; ILD=interstitial lung disease.

) (Forsythe and Faulkner, 2003b). Reasons for the difference in incidence between Japan and the rest of the world are unknown and require further scientific investigation. Interstitial lung disease has also been observed in patients who have received treatment with the epidermal growth factor receptor tyrosine kinase inhibitor erlotinib (Yamamoto *et al*, 2003).

The disparity of ILD reporting rates in Japanese patients with NSCLC compared with those in the rest of the world has raised some important questions involving epidemiology, disease mechanisms, disease comorbidity and methods of diagnosis. However, the demographic characteristics of gefitinib-treated patients with ILD reflected the characteristics of the overall patient population, and there was no obvious pattern to the ILD events with respect to time of onset, age, gender or previous treatments. Similarly, the comorbidities and previous/concurrent medications of patients treated post approval in Japan are similar to those of patients treated outside Japan on a compassionate-use basis as part of an Expanded Access Programme.

### Pathology of gefitinib-associated ILD

**Figure 2:**
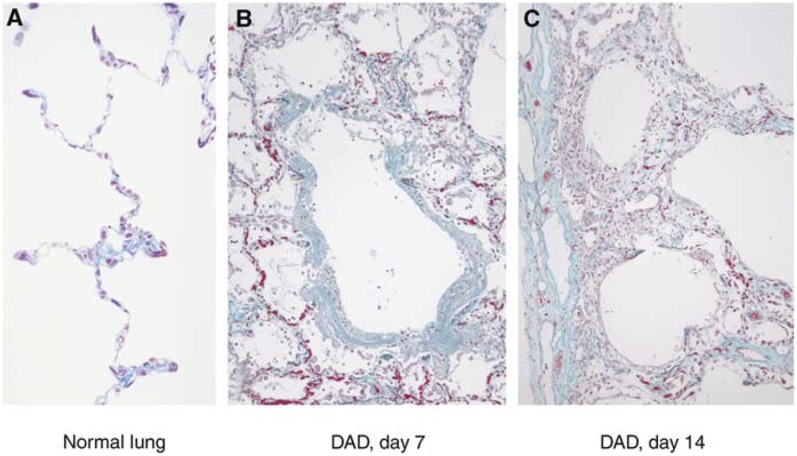
Pathology of gefitinib-associated ILD (AstraZeneca and Iressa Expert Committee, 2003).

In a series of eight patients who underwent autopsy following death from acute lung injury associated with the use of gefitinib, the pathological diagnosis of DAD was made (AstraZeneca and Iressa Expert Committee, 2003) (Figure 2

). Pre-existing ILD, including usual interstitial pneumonia, was found in three of the eight patients.

### Idiopathic pulmonary fibrosis as a prognostic risk factor for ILD associated with gefitinib

Clinical examination of the total population of 152 patients with ILD suggested that the presence of idiopathic pulmonary fibrosis (IPF) affected outcome from ILD (for an analysis of maximum likelihood estimates, *P*=0.048). There was pre-existing IPF in 23 out of 48 patients. For a group of 29 out of 152 patients for whom CT scans were available before and after onset of ILD, 12 had pre-existing IPF and 7 (58%) died; 17 did not have pre-existing IPF and only 3 (18%) of these patients died. Hence, the presence of IPF seems to be an important risk factor.

### Radiology of gefitinib-associated ILD

**Figure 3:**
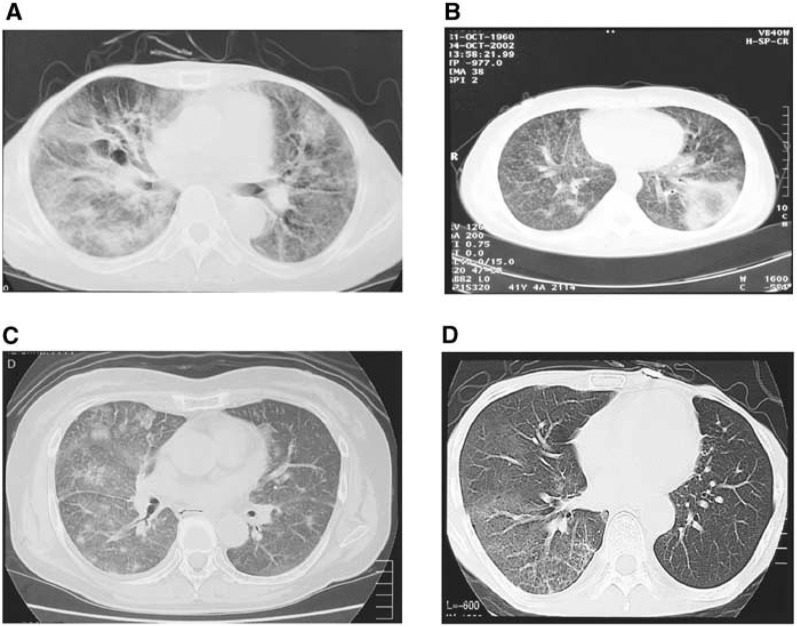
Radiology of gefitinib-associated ILD. (**A**) Acute interstitial pneumonia-like observations. (**B**) Chronic organised pneumonia-like observations. (**C**) Acute EP-like observations. (**D**) Light ground-glass shadows in bilateral lung fields lacking shrinkage of lung field and traction bronchiectasis (AstraZeneca and Iressa Expert Committee, 2003).

Clinical examinations of 152 patients with ILD were performed. In a series of 47 patients from this population for whom HRCT was available, the characteristic images of gefitinib-associated ILD were of patchy diffuse ground-glass shadows; these HRCT images were similar to those of drug-associated lung injury (AstraZeneca and Iressa Expert Committee, 2003). Several characteristic HRCT patterns that are seen in other drug-associated ILDs were also observed in the gefitinb-associated condition (e.g. acute interstitial pneumonia-like pattern, cryptogenic organised pneumonia-like pattern, EP-like pattern and localised infiltration pattern) (Figure 3

).

## CLINICAL EXAMPLES OF ILD ASSOCIATED WITH USE OF GEFITINIB

Case histories of four Japanese patients with NSCLC treated with gefitinib, who developed interstitial pneumonia during treatment at the Institute of Development Aging and Cancer, Tohoku University, Sendai, illustrate some of the clinical patterns and presentation of ILD in patients treated with gefitinib (Inoue *et al*, 2003). We present these case histories in greater detail here.

### First case

The first case described is that of a 69-year-old male smoker (15 pack-years) with stage IV bronchioalveolar carcinoma who had undergone three courses of chemotherapy. After 2 weeks' gefitinib treatment, an apparent reduction in the alveolar consolidation by bronchioalveolar carcinoma on HRCT scan was observed. However, on day 11 of gefitinib therapy, abrupt onset of high fever and subsequent dyspnoea was noted. New ground-glass attenuation (GGA) appeared in both sides of the upper lobes on HRCT scan. Serum levels of SP-D, KL-6, CRP and LDH were elevated. Serum SP-D and KL-6 are useful markers for alveolar damage in ILD.

Drug-associated interstitial pneumonitis was suspected and gefitinib therapy was continued for a further 2 months with concurrent administration of corticosteroids. By day 30, respiratory status was improved and the GGA of the upper lobes on HRCT scan was diminished. Gefitinib therapy ceased and corticosteroid therapy was continued for approximately 1 month until death due to bronchioalveolar carcinoma. At autopsy, there were no apparent lesions with DAD or evidence of acute lung injury and no infection was found.

### Second case

The second case described is that of an 85-year-old male smoker (50 pack-years) with squamous cell carcinoma of the lung. After radiotherapy (60 Gy), serum levels of both SP-D and KL-6 were gradually increased and GGA with reticular shadows was noted on HRCT scan, suggesting the onset of radiation pneumonitis. Furthermore, tumour size gradually increased after radiotherapy. Gefitinib therapy was started, but was discontinued on day 18 when sudden-onset anorexia and dyspnoea were noted. On day 20, acute lung injury was suspected due to acute onset of severe hypoxia and the bilateral diffuse distribution of GGA on HRCT scan. Death occurred 2 days later, in spite of high-dose steroid therapy.

At autopsy, squamous cell carcinoma tumours contained central necrosis and fibrosis, suggesting the effects of radiation and gefitinib therapy. Diffuse alveolar damage distributed chiefly on the right lung demonstrated marked congestion and oedema. The lesions with organising pneumonia were also densely distributed.

### Third case

The third case described is that of a 61-year-old male smoker (12 pack-years) with well-differentiated adenocarcinoma who had undergone right lower lobectomy 5 years previously and in whom metastases were noted in the left lung 2 years later. For 2 years various chemotherapy was administered but the tumour gradually enlarged.

Gefitinib therapy was started and continued for 83 days before being discontinued due to tumour progression. Treatment with gefitinib was restarted after 3 months along with one administration of docetaxel (60 mg m^−2^) after 11 days of gefitinib. At 19 days after gefitinib treatment was restarted, high fever, dyspnoea, hypoxemia and elevated serum levels of SP-D, KL-6 and CRP were observed. Antibiotic therapy was not effective and, due to bilateral GGA on HRCT scan and high SP-D and KL-6 levels, drug-associated pneumonitis was strongly suspected. After high-dose steroid therapy, symptoms and GGA on HRCT were remarkably improved.

### Fourth case

The fourth case described is that of a 78-year-old male smoker (63 pack-years) with stage IIIb large cell carcinoma who had undergone radiotherapy (66 Gy). Ground-glass attenuation noted on HRCT scan suggested radiation pneumonitis and steroid therapy was started. Treatment with gefitinib was stared while the patient was still receiving steroid treatment. On day 4 of gefitinib therapy, dyspnoea was noted and gefitinib was discontinued. Serum levels of CRP, SP-D and KL-6 were markedly elevated and gefitinib-associated ILD was suspected. Dyspnoea deteriorated in spite of high-dose steroid therapy, with abrupt increase of GGA on both sides of the lung; death occurred 5 days later.

At autopsy, a squamous cell carcinoma showing coagulation necrosis and haemorrhage in the lower lobe of the right lung was observed along with DAD showing hyaline membranes incorporated to collapse fibrosis with honeycomb appearance. Granulations similar to the organising pneumonia pattern and bronchopneumonia were also scattered. Immunohistochemistry demonstrated a limited number of the epithelial cells infected by cytomegalovirus.

## SUMMARY

Drug-associated ILD is common, with diverse patterns ranging from benign infiltrates to life-threatening acute respiratory distress syndrome. An accurate diagnosis of drug-associated ILD is based on clinical, radiological and histological manifestations. However, diagnosis is often only possible by exclusion. While ILD should be monitored carefully in NSCLC, the context of the overall toxicity profile of the treatment and the serious nature of the disease should be considered alongside the efficacy of the agents concerned. Standard chemotherapy regimens are associated with a degree of toxicity and toxicity-related death (Schiller *et al*, 2002) and, furthermore, patients with advanced NSCLC have a very poor prognosis, with few treatment options, and ILD is a comorbid disease of lung cancer. Gefitinib is an example of a new targeted therapy for the treatment of lung cancer that has demonstrated unprecedented activity in pretreated patients and is not associated with the burden of toxicity of chemotherapy. Cases of ILD have been reported in Japan for patients treated with gefitinib, with an overall incidence higher than that observed in the rest of the world. Although vigilance for pulmonary toxicity is mandatory, overall, the balance of clinical benefit with gefitinib outweighs the risk of ILD.
